# Development of chimeric antigen receptor-redirected T cell therapy targeting L1-CAM in ovarian cancer

**DOI:** 10.1186/2051-1426-1-S1-P16

**Published:** 2013-11-07

**Authors:** Hao Hong, Christine Brown, Stephen J Forman, Michael Jensen

**Affiliations:** 1 Beckman Research Institute, City of Hope National Medical Center, Duarte, CA, USA; 2 Seattle Children’s Research Institute, Seattle, WA, USA

## 

Ovarian cancer is responsible for the majority of gynecologic cancer deaths. Despite improvements in surgical approaches and the refinement of first-line cytotoxic combinations, the majority of patients diagnosed with advanced stage ovarian cancer eventually succumb to tumor recurrence. Thus, novel therapeutic approaches are desperately needed for this disease. With the growing recognition of the importance of immune modulation in ovarian cancer development, various immune-based modalities have been actively explored. Here we present a novel adoptive immunotherapy strategy that utilizes chimeric antigen receptor (CAR) engineered T cells reprogrammed to target L1-CAM. Our studies demonstrate L1-CAM is an ovarian tumor associated-antigen - it is over-expressed on a panel of established and primary ovarian cancer cell lines, as well as detected at various levels in the majority of the primary ovarian tumor specimens interrogated (39 of 40 cases). Importantly, L1-CAM was not expressed on normal ovary suggesting an excellent window opportunity for tumor specific targeting. We achieved HLA-independent L1-CAM targeting by engineering T cells to express a CAR, designated as CE7R, comprised of an scFv derived from the L1-CAM-specific monoclonal antibody CE7 linked to the CD28 co-stimulatory and CD3-zeta endodomains. We report that these genetically re-programmed CE7R+ T cells specifically and efficiently killed a panel of L1-CAM+ human ovarian cancer cells in vitro including OVCAR-3, MADH2744, Caov3, SKOV3; but did not kill L1-CAM-negative cell lines such as A2780. In concordance with the cytolytic assay, the CE7R+ T cells were activated to produce proinflammatory cytokines such as IFNγ, TNFα and GM-CSF upon co-culture with L1-CAM+ tumor cell lines, but failed to do so upon stimulation with L1-CAM-negative cells. Moreover, CE7R+ T cells also lysed primary cultured L1-CAM positive cancer cells derived from ovarian cancer patients’ ascites. Importantly, a single dose intraperitoneal administration of CE7R+ T cells induced regression of i.p. established OVCAR-3 xenograft tumor growth in mice. For the first time, these results demonstrate L1-CAM is amenable to targeting by CAR+ T cells and support the potential of this CAR-based adoptive T cell immunotherapeutic approach in treating ovarian cancer.

